# Using a Multi-Level Process Comparison for Process Change Analysis in Cancer Pathways †

**DOI:** 10.3390/ijerph17197210

**Published:** 2020-10-01

**Authors:** Angelina Prima Kurniati, Ciarán McInerney, Kieran Zucker, Geoff Hall, David Hogg, Owen Johnson

**Affiliations:** 1 School of Computing, University of Leeds, Leeds LS2 9JT, UK; C.McInerney@leeds.ac.uk (C.M.); D.C.Hogg@leeds.ac.uk (D.H.); O.A.Johnson@leeds.ac.uk (O.J.); 2 School of Computing, Telkom University, Bandung 40257, Indonesia; 3 School of Medicine, University of Leeds, Leeds Teaching Hospitals Trust, Leeds LS1 3EX, UK; K.Zucker@leeds.ac.uk (K.Z.); G.Hall@leeds.ac.uk (G.H.)

**Keywords:** process mining, cancer pathways, process change, concept drift, multi-level process comparison

## Abstract

The area of process change over time is a particular concern in healthcare, where patterns of care emerge and evolve in response to individual patient needs. We propose a structured approach to analyse process change over time that is suitable for the complex domain of healthcare. Our approach applies a qualitative process comparison at three levels of abstraction: a holistic perspective (process model), a middle-level perspective (trace), and a fine-grained detail (activity). Our aim was to detect change points, localise and characterise the change, and unravel/understand the process evolution. We illustrate the approach using a case study of cancer pathways in Leeds where we found evidence of change points identified at multiple levels. In this paper, we extend our study by analysing the miners used in process discovery and providing a deeper analysis of the activity of investigation in trace and activity levels. In the experiment, we show that this qualitative approach provides a useful understanding of process change over time. Examining change at three levels provides confirmatory evidence of process change where perspectives agree, while contradictory evidence can lead to focused discussions with domain experts. This approach should be of interest to others dealing with processes that undergo complex change over time.

## 1. Introduction

Process mining research projects, like other data analytics projects, work with data collected over months or years. Those projects are generally started by assuming that there no change occurred to the processes during the period of study. In reality, there is a high possibility that changes occurred in both the process and the data generated by that process. In healthcare, this is a particular concern because patterns of care emerge and evolve in response to individual patient needs, care procedures, and Electronic Healthcare Record (EHR) system changes, among other reasons. Those changes happen through complex interactions between people, processes, technology and changing organisational structures. It is important to identify and model process change over time in healthcare process mining so that intended changes can be monitored and unintended changes can be investigated and understood.

The term ‘concept drift’ has been used by the machine-learning community to describe the changing nature of processes over time [1]. Process mining community adopted this term to describe the dynamic nature of the process or the data recorded about the process. There is a growing body of literature exploring new approaches to analyse concept drift [2,3,4]. There are three challenges when dealing with concept drift: (1) change point detection, (2) change localisation and characterisation, and (3) change process evolution. Change point detection is about detecting a process change and localisation is about determining the period within which this change occurred. Change characterisation aims to understand the nature of a change and describe the elements of a process change. Change process evolution aims to understand the evolution of a process over time.

Process change analysis is commonly done by constructing process models from different time windows in a large dataset and comparing them to each other to identify process changes. This approach is related to process comparison, which has been widely applied for conformance checking, where a reference model is compared to the event log recording the real process execution [5]. A process comparison approach to process change analysis proposed by Partington et al. [6] included defining comparison points based on various metrics. However, Partington et al.’s approach was used to compare processes between different hospitals and is not directly applicable to process change analysis. Furthermore, Partington et al.’s approach required clinical understanding to select specific clinical metrics that vary between different clinical domains. Bolt et al. [7] proposed another process comparison approach based on the differences in activity frequencies and percentages in the logs. This approach facilitated a detailed comparison between each activity in two logs, but not directly between processes. Both works by Partington et al. and Bolt et al. are not directly related to process change analysis, but the underlying ideas of comparison can be used for analysing process change over time. While each of these approaches provide some insight into process change, there is no omnibus method that benefits from the advantages and perspectives of them all.

In this paper, we present an exploratory study where we discover and analyse changes over time in complex longitudinal healthcare data. Our aim was to adopt a multi-level approach to detect, localise and characterise process change. Our case study was drawn from the EHR system of the Leeds Teaching Hospital National Health Service Trust (LTHT). The LTHT hosts one of the UK’s largest cancer centres (Leeds Cancer Centre). We examined process data on the routes to diagnosis of endometrial cancer patients over 15 years (2003–2017). Our previous literature review found that process mining has been used in oncology and shows promising results to support process analytics [8]. In earlier work with the Leeds Cancer Centre data [9,10], we had assumed that there were no changes to the process during the time period. We were aware that the period was long, that the organisation, system, and people had evolved and changed over time, but we were not aware of specific process changes before we commenced the study. However, our previous experience process mining MIMIC-III, an open-access database [11,12], showed that a system change can affect the discovered process. We were fortunate to have access to the Leeds EHR system developed in-house, Patient Pathway Manager (PPM) [13,14], along with access to the parties involved in the treatment process: clinical staff, senior clinicians, the training team, and the software developers of the PPM system. Our multi-level process change analysis method [15] detected changes that can be explored and discussed with those parties to identify potential causal links between changes detected in the data and in practice.

## 2. Materials and Methods

### 2.1. General Methodology of the Multi-Level Process Change Analysis

**Figure 1:**
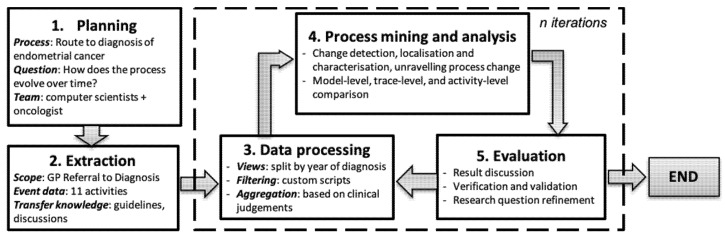
The general methodology.

Our general method combined the well-established Process Mining Process Methodology (PM^2^) [16] with concept drift analysis [4]. A process change analysis proposed by Bose et al. [4] suggested three steps: process change detection, localisation and characterisation, and unravelling process evolution. We proposed an approach to apply those steps through a process comparison at three different levels: process model, trace, and activity levels. We used our approach to analyse a case study of the route to the diagnosis of endometrial cancer. Our general methodology is shown in Figure 1.

As presented in Figure 1, *planning* defined the business process of interest, the research questions, and team members involved in this study. Our study focused on the route to diagnosis of endometrial cancer. Our research question was “How does the process evolve over time?”. Our team consisted of computer scientists and oncologist. *Extraction* included steps to select the 11 events recorded as part of a patient’s process from referral by a General Practitioner (GP referral) until the endometrial cancer diagnosis. In *Data processing*, we split the event log into sub-logs consisting of traces of patients based on the diagnosis year. Some filtering and aggregation were conducted based on clinical experts’ judgements. *Process mining and analysis* applied three steps of process change analysis at three different levels of process comparison. Finally, the *Evaluation* stage involved discussion, verification and assessment of the validity of the findings, with clinical experts.

The main components of our study can be expressed in the following definitions.

**Definition 1** **(Event logs and traces).** An event log, **E**, is a set of events described by a case_id, an activity name, and a timestamp, (**c**, **a**, **t**). An event describes an activity, **a**, which happened in a particular case, **c**, at a timestamp, **t**. A trace, **T**, is a temporal sequence of events that occurred in a case, where **T** ϵ **E**. In this study, a case represents a patient who has a set of events between the timestamps of referral and diagnosis of endometrial cancer.

Table 1 shows a fictitious but illustrative event log similar to the event log used in this study. This event log consists of the timestamped events of two patients. The first patient (P001) had four recorded events that can be written as a trace (referral–investigation–multi-disciplinary team (MDT) review–diagnosis). The second patient (P002) had five events that can be written as a trace (referral–outpatient–MDT review–diagnosis–surgery).

**Definition 2** **(Sub-logs).** A sub-log, **S**, is a partition of an event log, **E**, based on partitioning criteria. The partitioning should be done in such a way that a trace is grouped into a sub-log with no duplication between sub-logs. In this study, the event log was divided into sub-logs based on the year of diagnosis of each patient. There are clearly many partitioning options that could be adopted.

**Figure 2:**
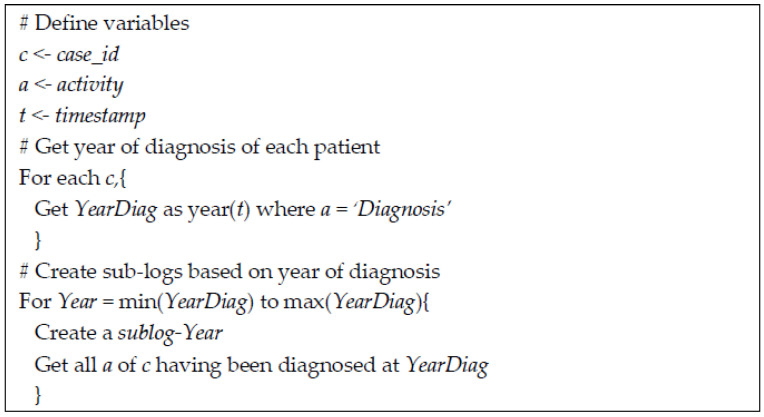
The algorithm of sub-log creation.

Figure 2 shows the algorithm used in this study to create sub-logs. The year of diagnosis was used because the diagnosis was the starting point in the selection criteria of patients included in this study.

**Definition 3** **(Process models).** A process model, **M**, is a directed graph representing the traces, **T**, in the event log, **E**. A process model consists of nodes representing activities, **a**, and arcs representing the possible paths, **p**, from one activity to another. A process model can be discovered using standard process mining algorithms. A process model might have additional components, such as the percentage of **a** and **p** to all activities and paths in **E**, respectively.

### 2.2. Process Change Analysis

The analysis of the sub-logs was based on selected metrics at the process model, trace, and activity levels to describe the multi-level behaviour of the processes of interest. A summary of the metrics used in this study is presented in Table 2. 

For the model-level comparison, we investigated the performance of four miners in ProM 6.8. Those four miners are Integer Linear Programming (ILP), the interactive Data-Aware Heuristics Miner (iDHM), the Inductive Miner (IM), and the Inductive Miner—Infrequent (IMf). These miners represent discovery algorithms from three different approaches. ILP represents region-based miners that construct a Petri net from a description of the behaviour in a transition system [17]. The iDHM represents heuristics-based miners that use a process-representation language in causal nets [18]. The IM and IMf represent two variants of inductive-based miners that construct a process tree to guarantee re-discoverability [19]. The IM discovers a complete graph, while IMf excludes infrequent traces. For all four miners, we used the default parameters in ProM since parameter optimisation is outside the scope of this paper. The general process model was built from the complete event log from 2003 to 2017. The model-level behaviour was described by the replay fitness, precision and generalisation [5] of each sub-log (i.e. each calendar year) to the general model. 

The trace-level behaviour was described by the durations and variants of the traces in the sub-logs using bupaR [20]. The duration of referral to diagnosis was analysed further in relation to the new 28-day waiting time target as set by the Cancer Task Force. Trace variants were explored in more detail to analyse types of investigations undertaken during patient treatment.

The activity-level behaviour was described by activity frequency and its percentage in the sub-logs. A visual–analytic approach was adopted to identify possible changes by comparing the visualisations of these descriptions of process behaviour. The comparison was done quantitatively. As a follow-up on the deeper analysis of the investigation events, we explored different types of investigations in the activity-level comparison.

### 2.3. The Route to the Diagnosis of Endometrial Cancer: A Case Study

Our previous literature review found that process mining has been used in oncology and shows promising results to support process analytics [8]. We reviewed the case studies examined in publications and found that there were only 14 case studies between 2008 and 2016 and they were dominated by a well-prepared dataset in the Business Process Intelligence Challenge (BPIC) 2011. This suggested the lack of available datasets for process mining in oncology. General complaints about providing real case studies arise because of limited access to hospitals, difficulties in granting access due to confidentiality and ethical concerns, and the limited number of cases in oncology. Our case study was based on a real dataset extracted from the PPM EHR system of the LTHT as a large cancer centre with the additional advantage of direct access to expertise in the hospital. Another extract from the same database has been used in a previous study [9] where we described the provenance and characterisation of the database. We explored and made sure that this dataset contained the required components for process mining and thus was suitable to be used in this study.

Our case study focused on the route to the diagnosis of endometrial cancer of Leeds patients over an extended fifteen-year time period. Endometrial cancer is one of the most common cancers in the gynaecology department, and it was understood that the procedure for endometrial cancer treatment had not been changed radically within the last fifteen years. We used an event log from the PPM EHR that has been anonymised and cleaned, as documented in full in [9]. We extracted events from GP referral to the diagnosis of endometrial cancer. One of the important performance indicators in cancer treatment is the route to diagnosis [21] or cancer waiting time [22]. In the UK, the performance of cancer waiting time is monitored by Public Health England and forms a key benchmark for high-quality cancer care. In 2015, the Cancer Taskforce recommended the introduction of a new 28-day faster diagnosis standard. Changes to the cancer waiting time system and dataset were introduced from April 2018 to mark the start of the implementation of this new standard, which was fully implemented in 2020. Even though our data are on 2003–2017 cohorts, we could use our cohorts to see if the historical data would fit into the new standard.

Endometrial cancer is a common cancer in women who have been through the menopause. This cancer affects the female reproductive system with the most common symptom being unusual vaginal bleeding. This symptom is usually checked in a GP consultation and then the patient is referred to an oncology specialist (gynaecologist). The specialist conducts some tests (investigations), such as an ultrasound scan, a hysteroscopy or a biopsy. The results of the investigations are then discussed in a multi-disciplinary team (MDT) review to gain a consensus from clinical experts. If diagnosed with endometrial cancer, the cancer stage will be defined by further investigations [23].

All of these events have been coded and recorded in the PPM EHR at the Leeds Cancer Centre since 2003. The extracted event log should therefore consist of those records and is a rich data source for process mining of the pathways of interest. The PPM EHR consists of at least 50 tables, including nine tables relevant to our study of the pathways from GP referral to an endometrial cancer diagnosis. Those nine tables contain broad categories of activity, such as referrals, outpatients, and diagnosis.

## 3. Results

### 3.1. Data Extraction and Data Processing Stages

We created the study cohort by applying four selection criteria. Patients were included in the study if they had:

- a legitimate care relationship with the LTHT,
- a primary diagnosis of endometrial cancer (ICD-10 code C54 or C55),
- an endometrial cancer diagnosis between 2003 and 2017,
- been diagnosed at a maximum of 120 days after referral to an oncology specialty.

The last criterion was decided based on discussions with clinical experts who argued that a referral period greater than 120 days is implausibly long for the events to be related. By applying those four selection criteria, we selected 943 out of 1126 endometrial cancer patients (84%). There were 65,200 events selected based on all patients or 58 events per patient on average.

We extracted all time-stamped events between GP referral and diagnosis from the selected patients. This step resulted in 339 different activity types. For our study, we focused simply on the broad categories of activity represented by the nine tables. We separated admissions and discharges from their combined table and separated diagnostic surgery events from the surgery table. The resulting 11 activities agreed with the prior knowledge of our clinical co-authors. Those activities are presented in Table 3 along with the number of occurrences, the number of patients and the percentages. The percentage of the occurrence was calculated against the total number of records. The percentage of patients was calculated by the total number of patients rather than records and so did not add up to 100%. The event log contained 7967 events with a minimum of 2, a median of 6, a mean of 6.3, and a maximum number of 12 events per patient.

### 3.2. Process Model Comparison

Table 4 shows the replay fitness, precision and generalisation of the four miners evaluated. We used the four miners with the default parameters in ProM and represented the maximum number of traces and activities. The ILP showed perfect precision and high generalisation, but had the lowest replay fitness. This means that the ILP discovered a model that is highly generalisable and precise, but does not fit a high percentage of the individual traces. The IM showed an optimal replay fitness, but low precision and zero generalisation. The IM focused on discovering a model that is fitted for all traces in the event log, but is not precise nor generalisable. The IMf is better than IM for precision, worse for replay fitness, but similarly poor for generalisability. Both IM and IMf have 0.00 generalisation, which suggests that both models are not generalisable for future behaviours. The iDHM process model showed a model with high replay fitness, precision, and generalisation. While ILP and iDHM both performed well, we favoured iDHM because ILP’s precision of 1.00 suggests an overly specified model while performing relatively poorly for replay fitness.

**Figure 3:**
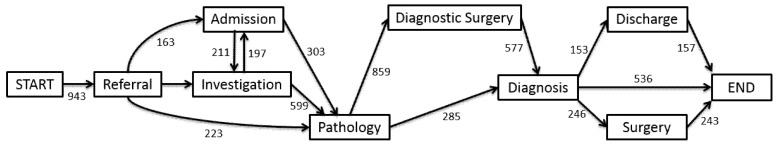
The Data-Aware Heuristics Miner (iDHM) process model. Originally produced by the iDHM plugin in ProM 6.8, showing the process model of the pathway from referral to diagnosis. The pathway flows from left to right, with rectangles representing activities and arrows as flows from one activity to the other. The numbers on the arrows show the number of patients with activity flows to other activities.

The general process model showed in Figure 3 is based on iDHM of the complete log for the full 15 years. For simplicity, we present the process model of the eight most-frequent activities and the most-frequent paths between them. The infrequent activities that were omitted from the process model are outpatient, consultation, and MDT review. The outpatient activity appeared in 149 out of 943 patients (16%), consultation appeared in 152 out of 943 patients (16%), and MDT review appeared in 231 out of 943 patients (24%).

**Figure 4:**
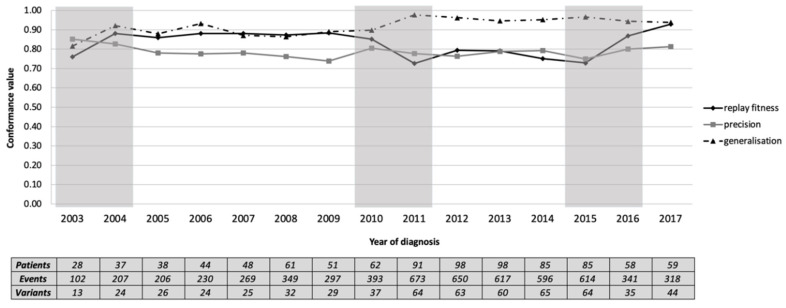
Annual conformance to the general process model. The shaded areas show the periods where change might have occurred at the process model level.

The event log was divided into 15 sub-logs based on the year of diagnosis. We then tested each sub-log for conformance against the general process model. The model remained reasonably representative of each yearly sub-log (median [inter-quartile range]: replay fitness = 0.86 [0.10], precision = 0.78 [0.03], and generalisation = 0.93 [0.06]). Figure 4 shows that all measures were similar throughout the years. Our qualitative assessment suggested three candidate changes: 2004, based on the increased replay fitness and generalisation; 2011, based on a drop in replay fitness and increase in generalisation; 2016, based on the increased replay fitness and precision. The three identified periods of potentially significant change are 2003–2004, 2010–2011 and 2015–2016.

### 3.3. Trace Comparison

**Figure 5:**
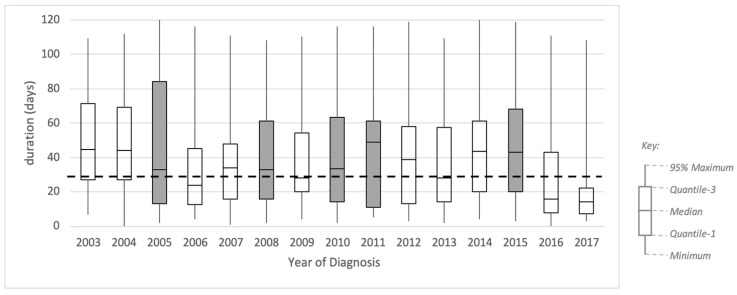
Boxplot of number of days from GP referral to diagnosis by year of diagnosis. Dashed line shows target duration (28 days). The shaded boxes show the periods where change might have occurred.

Figure 5 shows a box plot representing the profile of trace duration from referral to diagnosis for each yearly sub-log. The box plot shows no obvious qualitative pattern based on the distribution of the duration, except on the inter-quartile range (IQR). The IQR is generally decreasing throughout the years, with some exceptions. Based on these results, we identified five candidate periods of change: 2005, where the IQR increased from 42 to 71 days (68%); 2008, where the IQR increased from 32 to 45 (39%); 2010, where the IQR increased from 34 to 49 days (44%); 2011, where the IQR increased from 49 to 50 days (2%); 2015, where the IQR increased from 41 to 48 days (18%). 

Additionally, in Figure 5, we included a dashed line showing a target duration of 28 days. This duration is the new waiting time target set by the Cancer Task Force in 2015. This new target was introduced from April 2018 and was fully implemented in 2020. Even though our data are on the 2003–2017 cohort, we show this target to analyse whether the historical data would fit into the new standard. It is shown in Figure 5 that the median durations in 2009 and 2013 were fit to the targeted duration, and 2017 was the first time that the year’s interquartile range was below the targeted duration. 

**Figure 6:**
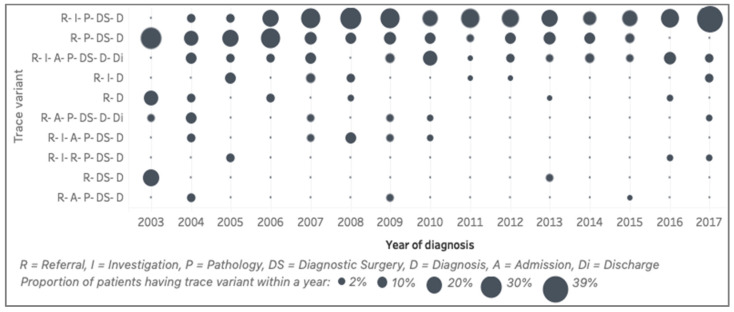
Summary of the trace variant comparison (2003–2017). Size represents the percentage of trace variants over the number of patients diagnosed in each year.

The top ten trace variants (representing 52%) of the general model and the presence of those variants over the years are shown in Figure 6. Those top ten trace variants show only seven activities, excluding surgery. The first variant is the sequence of referral (R), investigation (I), pathology (P), diagnostic surgery (DS) and, finally, a diagnosis (D) of endometrial cancer. This variant is the most-common pathway of the patients in the cohort. The second variant is similar to the first one, but with no record of investigation. We discussed this with the clinical experts and found that this reflected a process change in the EHR system, where investigation had not been recorded in the system in the early years. The third variant is similar to the first, except that the patients were admitted after an investigation. The three most common variants (median [IQR]) are R–I–P–DS–D (19 [9]%), R–P–DS–D (10 [7]%), and R–I–A–P–DS–D–Di (6 [3]%). The qualitative distinction between the years is the wavering trend of the first variant and the decreasing trend of the other variants. For example, variant 2 (R–P–DS–D), variant 5 (R–D) and variant 9 (R–DS–D) were frequent in 2003, but became infrequent in later years.

### 3.4. Activity Comparison

**Figure 7:**
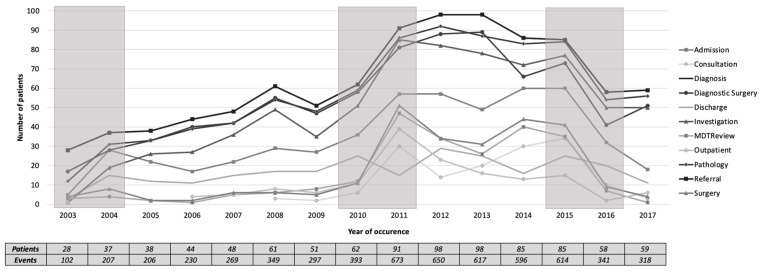
The total number of patients undergoing each of the main activities (2003–2017). The shaded areas show the periods where change might have occurred at the activity level.

Figure 7 shows a plot of the total number of patients undergoing each of the main activities over the years. In 2003–2004, there was a sudden increase in almost all activities. In 2010–2011, there was a sudden increase in almost all activities except on discharge. Meanwhile, in 2015–2016, there was a sudden decrease in all activities. These three periods of change were also suggested in Section 3.2 when reviewing the model level.

**Figure 8:**
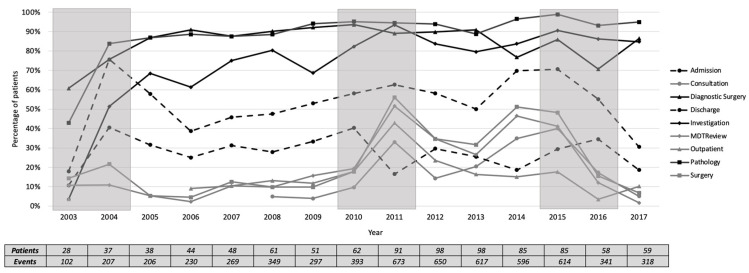
Percentage of activity presence by the number of patients each year. The solid black lines represent frequent activities; the solid grey lines represent infrequent activities and highly varied activities. The shaded areas show the potential change periods at the activity level. Referral and diagnosis occurred in 100% of patients and are not presented for simplicity purposes.

We plotted the percentage of each activity for the number of patients each year, as presented in Figure 8. The activities were grouped into frequent activities, occurring in at least 60% of patients, infrequent activities, occurring in less than 60% of patients, and highly varied activities in between. The three most frequent activities in all years (median [inter-quartile range]) are pathology (93 [7]%), diagnostic surgery (87.5 [9]%), and investigation (80 [16]%). The four most infrequent activities are MDT review (12 [20]%), outpatient (13 [7]%), surgery (15.5 [23]%), and consultation (16 [24]%). Qualitatively, the period of 2010-2011 was marked by a change in the frequency of the four infrequent activities, while Discharge was decreased to be lower than the four infrequent activities. In 2013, the frequency of the infrequent activities was increased, except for outpatient.

Our discussion with clinical experts suggested that investigation is the most-important activity in the pathway of referral to the diagnosis of cancer. We then analysed the investigation by examining the occurrence and the number of patients undergoing different categories of investigation. Our clinical experts (K.Z. and G.H.) categorised the investigation labels into 17 categories. 

Table 5 shows a summary of the categorisation, the occurrence and the number of patients undergoing the investigation categories. It is shown that the most frequent category is unknown (498/34%), which shows the nature of incomplete records in the EHR system. It is followed by diagnostic sampling, ultrasound scans (USS), MDT, plain film, MRI, and CT. The ten other categories occurred less than five times in the event log, leaving only six frequent categories to analyse. Those six frequent categories are diagnostic sampling, USS, MDT, plain film, MRI, and CT.

**Figure 9:**
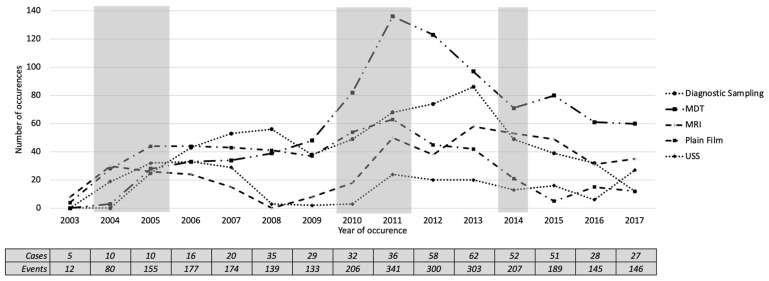
The total number of patients undergoing each of the five most frequent investigation categories (2003–2017). The shaded areas show the periods where change might have occurred at the activity level.

Figure 9 shows a plot of the number of occurrences of the five most-frequent investigation categories over the years. We found five periods where changes might have happened, based on the occurrence of investigation categories. In 2004, all investigation categories except diagnostic sampling had increased with a mean increase of 13.6. In 2005, all investigation categories were increased with a mean of 15, except MRI, which was slightly decreased from the previous year. In 2010 and 2011, all investigation categories were increased with averages of 14.6 and 27, respectively. In 2014, all investigation categories were decreased, with an average of 19. These candidate changes in 2004, 2010 and 2011 agreed with the candidate changes identified by the activity presence (Figure 8).

### 3.5. Additional Analysis

We conducted the same analysis of the trace-level comparison to examine the 17 investigation categories. This additional analysis can be seen as a way to analyse the process at a different level of granularity. A summary of the top 10 trace variants with the occurrence and percentage is shown in Table 6.

Table 6 shows that the top ten trace variants from the general model represented 44.7% of all traces and included only five types of Investigation, which were USS, MRI, MDT, diagnostic sampling, and plain film. The low number of traces represented in the top ten trace variants imply the high variability of the sequence of investigation categories undertaken by the patients. Of all 768 cases with recorded investigations, there are 182 trace variants (24%). The most-common trace variant is MRI–MDT (7.3%), followed by the second trace variant MDT–diagnostic sampling (6.8%) and the third trace variant is MDT–plain film–diagnostic sampling (5.9%).

## 4. Discussion

We analysed our case study of endometrial cancer care in Leeds Cancer Centre following the PM2 methodology. We focused on the first five stages of PM2 (excluding process improvement and support) to exemplify the appropriate application of our novel, multi-level, exploratory approach. Our approach supports the exploration for unknown process changes through a qualitative, multi-level perspective for detecting, localising and characterising the changes in processes. Graphical data visualisations make use of humans’ natural pattern-seeking capacities and support discussions with domain experts about process evolution and change. We found that such discussions helped us to reflect on the nature of the process change over time and to generate hypotheses about potential causal links between changes detected in the data and changes in practice. 

These hypotheses can then be tested by further, more tightly focused process and data analysis. In that respect, our approach is perhaps just a starting point for further exploratory studies. In this case study, we split a fifteen-year event log into 15 yearly sub-logs. Further analysis can be done to examine the division of the log into months, weeks, days or hours to isolate potential change events of interest. It is important to note that the shorter the duration of each division will lead to a smaller number of traces in each division, which could potentially lower the confidence in the results.

We based our approach on Bose et al.’s process change analysis: detection, localisation and characterisation, and unravelling [4], and Partington et al.’s process comparison [6]. Bose et al. suggest that the best place to start analysing process change is by detecting that a process change has taken place. If that is the case, then the next steps are to identify the time periods at which changes happened and characterise the nature of the change. As an improvement to Bose et al.’s approach, we detected the changes from multiple levels, rather than just at the activity level. The process comparison approach described by Partington et al. requires the domain expert to pre-characterise the expected differences that they want to detect and localise. In contrast, our approach did not require prior specification of the changes. Instead, we used domain experts in the later stage of what Bose et al. describe as “Unravelling”. This approach supports initial exploration without over-burdening domain experts or when they are not able to pre-specify the expected differences.

### 4.1. Change Detection

Changes were detected at all three levels: five at the trace level and three at model and activity levels. From our blinded exploration of our case study process, we cannot attest to the validity of these detections nor can we know about any true process changes that were not detected. Our approach visualised the trend over time in three different levels of comparison and presented those visualisations to the experts. We approached the change detection through discussions with clinical experts and members of the developer teams to identify several possible changes in the clinical and technical aspects that might affect the changes evidenced in the data. Future work using simulations could attempt to determine the sensitivity of our suggested approach to changes in the magnitude and characteristics of process changes.

### 4.2. Change Localisation and Characterisation

With assumptions that the detected changes were true changes, we localised the changes to the 2010–2011 and 2015–2016 periods. These localisations were supported by an agreement between changes in metric values across multiple levels. Our multi-level approach shows its usefulness in the sense that the changes detected at one level can guide a focused investigation of the same period at other levels. For our case study, despite detection at the model and activity level, no change was independently detected at the trace level in the 2003–2004 period. There are also possible changes in 2005 and 2008 based on the duration analysis, where the IQRs of durations were increased. A review of the trace durations led us to consider that the median duration in 2016 was the first example of a year’s median duration lying outside the interquartile range of the previous year. Nevertheless, agreement in the localisation of changes supports the confidence of the change detection.

The aforementioned post-hoc review of trace durations is an example of how our approach facilitates change characterisation at levels where changes would not be found if levels were investigated in isolation. The change characterisations of a level can provide evidence to support or refute the characterisations of the other levels. The different median duration of traces was the only trace-level characterisation of change in 2016. The strongest evidence for a 2016 change came from the sharp decline in trace frequencies at the activity level and was supported by an unexpected rise in replay fitness at the model level. This multi-dimensional perspective of a suspected change event informs a more-rounded, complex picture of changes that can be taken to domain experts for discussion.

### 4.3. Unravelling Process Evolution

The rich characterisation stage provides the substance for a discussion with clinical experts as we explain the mechanism and consequences of the change detection. Our discussion with clinical experts to unravel process evolution found several findings based on three different levels of process comparison. The process model discovered (Figure 3) has been agreed to reflect the simplified general pathways from referral to the diagnosis of endometrial cancer. The perspective of the trace-level comparison (Figure 5; Figure 6) confirmed that there was no significant change in the duration and sequence from referral to diagnosis that the clinical experts were aware of. Clinical experts raised concerns about some trace variants (Figure 6) that have an admission (A) without a discharge (Di) (variants 7 and 10). Further analysis revealed that the discharge was not captured in the trace variants because the discharge event happened after diagnosis. In the activity level comparison, clinical experts highlighted their concerns regarding the finding that MDT review is one of the infrequent activities, while, in fact, all patients would need to be discussed in an MDT review at some point during their cancer treatment. Further discussions revealed that MDT reviews usually happen after a cancer diagnosis. We learnt from these concerns that the definitions of the start and endpoints of the pathways are crucial in defining what will be represented in the process model.

Another important discussion about the activity-level comparison is that the findings reflected the evolving nature of the EHR system. For example, it is shown in Figure 6 that the outpatient activity started to appear for patients diagnosed in 2006 and the consultation activity started to appear for patients diagnosed in 2008. At the more fine-grained level of investigation, the MDT and USS categories started to appear for patients diagnosed in 2004, while diagnostic sampling started in 2005. These evolutions were confirmed in a further discussion with the PPM development team, where we learnt that the system was modified in these years to start recording these respected activities. There were also improvements to the PPM system that introduced automatic imports from other systems (pathology, for example) that were previously captured manually. When the system was improved, the data volume and the reliability of the data increased. There are opportunities for further analysis to examine the effect of these changes in the system on the process over time.

### 4.4. Additional Analysis

Our additional analysis focused on the different types of investigation undertaken during patient treatment. This was motivated by the initial finding that investigation was one of the most frequent activities in the event log. Our clinical experts confirmed that a patient might have more than one type of investigation and the choice of the most appropriate type of investigation required a complex examination of patient conditions. We started our analysis by categorising the 53 different types of investigation into 17 categories. We checked the occurrence of those 17 categories in the event log and found that there were five frequent recurring categories within them. Those investigation categories were then used in the deeper comparison of both the activity and trace levels over the years. This additional analysis plays a role as an example of how our multi-level approach can be further expanded to a deeper analysis of a specific part of the process of interest. In our case study, we expanded our analysis to a deeper analysis of the investigation categories provided by the clinical experts. 

### 4.5. Reflection on Our Proposed Approach

We conducted an analysis to compare the process models created using the four most-commonly used miners in ProM 6.8. We expected to see that the general model was similar between miners, but we saw otherwise. The resulting models are varied based on the different approaches followed by different miners. We decided to discover our general process model using the iDHM plug-in [18], but another option, being entirely informed by clinical guidance, might have produced different process models. We used iDHM because its heuristic approach allowed us to obtain a process model at the desired level of detail. The visualisations provided in the plug-in made it possible to explore the directly-follows graph, dependency graph, causal net, or Petri net. We started our process model discovery by comparing models discovered using four different miners, which are ILP, IM, IMf, and iDHM. We found that iDHM performed well with a balanced quality of replay fitness, precision, and generalisation.

Another advantage evident in our approach is the splitting of the event log into sub-logs. We created sub-logs based on the calendar year of diagnosis. It is equally reasonable to split the log to enforce a uniform number of traces in each sub-log. The consequence of the first method is that the number of traces in each sub-log varies and the consequence of the second is that the duration of the sub-log varies. We addressed the consequence of our choice by analysing the frequency as a percentage instead of the number of occurrences. This splitting approach is needed in blind cases where there is no prior knowledge about any changes in the process. Otherwise, if there is a known process change, the event log should be split into before and after change sub-logs.

## 5. Conclusions

This paper presented a multi-level approach to analyse process changes over time and showed its applicability through a real-life case study. To the best of our knowledge, this is the first attempt to leverage the advantages and perspectives of existing process change methods that have historically focused on individual levels only. Our case study examined the pathways from GP referral to a diagnosis of endometrial cancer in Leeds Cancer Centre patients. The event log generated in this case study consists of events over a fifteen-year duration. It is representative of common EHR data with a long study duration. The general method followed in this study is PM2, with a focus on the process analytics stage to analyse process changes over time. Process change detection, localisation, and characterisation were carried out at three different levels of comparison: model, trace and activity. This approach allows for the detection of changes when comparing one year with another and answered the research question of this study: How does the process evolve over time? The change period detected and characterised in this study provided the substance for discussions with clinical experts and software development experts to unravel the process evolution. One important limitation of the proposed approach is that it is not able to detect the exact point in time when the change actually occurred. Moreover, this approach cannot detect changes back and forth during the same year. However, repeating the method with a finer-grained time interval would allow the change point to be more accurately detected, with the risk of having a smaller number of traces in each interval. Our additional analysis showed that we could work out the granularity level of the analysis to examine the pathway at a deeper level and focus on details of interest. For example, we examined investigations by inspecting them at a lower level of granularity to analyse pathways of interest in more depth.

Future work could review the splitting method, the comparison metrics, the reference model discovery, and the case complexity. The splitting method in this study was based on the calendar year of diagnosis, but other methods might be suitable in other cases, such as splitting traces into a uniform number in each sub-log or splitting based on a known change in interest. The comparison metrics used in this study are defined to represent three different levels of detail, but further work might examine other metrics for comparisons. The reference model discovery could be improved by considering clinical guidelines in the reference model, or by including only valid traces in the discovery step. This approach will also need to be tested on cases with different levels of complexity.

## Figures and Tables

**Table 1:** An illustrative event log.

Case_ID	Activity	Timestamp (YYYY-MM-DD)
P001	Referral	2020-01-06
P001	Investigation	2020-01-13
P001	MDT Review	2020-01-17
P001	Diagnosis	2020-01-31
P002	Referral	2020-01-21
P002	Outpatient	2020-01-22
P002	MDT Review	2020-01-31
P002	Diagnosis	2020-02-10
P002	Surgery	2020-02-10
…	…	…

MDT = Multi-Disciplinary Team.

**Table 2:** Metrics for multi-level process analysis.

Level	Metrics	Description
Model	Replay fitness	A measure of how many traces *T* from the sub-log *S* can be reproduced in the process model *M*, with penalties for skips and insertions; range 0–1.
	Precision	A measure of how ‘lean’ the model *M* is at representing traces *T* from the sub-log *S*. Lower values indicate superfluous structure in the model *M*; range 0–1.
	Generalisation	A measure of generalisability as indicated by the redundancy of nodes in the model *M*. The more redundant the nodes, the more variety of possible traces *T* that can be represented; range 0–1.
Trace	Duration	The number of days of trace *T* from *Referral* to *Diagnosis*.
	Variant proportion	The proportion of variants in the sub-log *S* that were one of the most frequent variants in the complete log *E*.
Activity	Frequency	The number of cases *c* undergoing an activity *a* in the sub-log *S*.
	Percentage	The percentage of cases *c* undergoing an activity *a* out of all cases in a sub-log *S*.

**Table 3:** The activity list.

#	Activity Name	Occurrence (%)	Patients (%)
1	Referral	943 (12)	943 (100)
2	Diagnosis	943 (12)	943 (100)
3	Investigation	1455 (18)	891 (94)
4	Diagnostic Surgery	1025 (13)	797 (85)
5	Pathology	1196 (15)	540 (57)
6	Admission	661 (8)	285 (30)
7	Discharge	581 (7)	193 (20)
8	Consultation	346 (4)	128 (14)
9	MDT Review	338 (4)	199 (21)
10	Surgery	248 (3)	234 (25)
11	Outpatient	231 (3)	135 (14)
Total		7967 (100)	

**Table 4:** Summary of process model quality.

Miner	Replay Fitness	Precision	Generalisation
ILP	0.72	1.00	0.99
IM	1.00	0.46	0.00
IMf	0.92	0.92	0.00
iDHM	0.81	0.83	0.99

ILP = Integer Linear Programming; IM = Inductive Miner; IMf = Inductive Miner-Infrequent; iDHM = Interactive Data-Aware Heuristics Miner.

**Table 5:** Summary of investigation categories. Occ (%) shows the number of occurrences and the percentage of the total occurrences in the event log. N (%) shows the number and percentage of patients undergoing a specific investigation category.

#	Category	Investigation Label	Occ (%)	N (%)
1	Unknown	Unknown	498 (34)	441 (57)
2	Diagnostic Sampling	Aspiration of lesion of breast, biopsy of lesion of organ ‘Not Otherwise Classified’, gynae cytology, non-gynae cytology, Pleural aspiration ± biopsy	233 (16)	218 (28)
3	USS	Doppler ultrasound, endoscopic ultrasound, transabdominal ultrasound, transvaginal ultrasound, ultrasound	214 (15)	210 (27)
4	MDT	Films reviewed at MDT meeting	184 (13)	146 (19)
5	Plain Film	Chest X-ray, sinus X-ray, skeletal survey	136 (9)	117 (15)
6	MRI	MRI -con, MRI (con unknown), MRI +con	107 (7)	106 (14)
7	CT	CT -con, CT (con unknown), CT +con, CT colonoscopy virtual	58 (4)	55 (7)
8	Investigative Surgery	Investigative surgery	4 (0.3)	4 (0.5)
9	Screening	Mammogram	4 (0.3)	4 (0.5)
10	Vascular Imaging	Angiography, angioplasty, Magnetic Resonance Angiography, Magnetic Resonance Venogram, venogram	4 (0.3)	4 (0.5)
11	Clinical Examination	Clinical examination	3 (0.2)	3 (0.4)
12	Bone density	Bone densiometry DXA	2 (0.1)	2 (0.3)
13	Nuclear Medicine	NMI, PET	2 (0.1)	2 (0.3)
14	Other	Other	2 (0.1)	2 (0.3)
15	Urine Sample	Urine	2 (0.1)	2 (0.3)
16	GI Imaging	Ba enema, barium enema, barium swallow, gastrografin enema, gastrografin swallow, MRCP, proctogram	1 (0.1)	1 (0.1)
17	Nuclear Medicine Imaging	Bone scan	1 (0.1)	1 (0.1)

-con = without contrast dye, +con = with contrast dye.

**Table 6:** Summary of the top ten trace variants based on the investigation categories.

#	Trace Variants	Occurrence (%)
1	MRI–MDT	56 (7)
2	MDT–Diagnostic Sampling	52 (7)
3	MDT–Plain Film–Diagnostic Sampling	45 (6)
4	MRI–MDT–Diagnostic Sampling	39 (5)
5	MDT	36 (5)
6	Diagnostic Sampling	30 (4)
7	MRI–MDT–Plain Film–Diagnostic Sampling	24 (3)
8	Plain Film–Diagnostic Sampling	21 (3)
9	MRI	20 (3)
10	USS–MRI–MDT–Diagnostic Sampling	20 (3)
Total		343 (44.7)
